# What drives the decoupling progress of China’s civil aviation transportation growth from carbon emissions? A new decomposition analysis

**DOI:** 10.1371/journal.pone.0282025

**Published:** 2023-03-06

**Authors:** Xiao Liu, Yancai Zhang

**Affiliations:** 1 College of Economics and Management, Huaiyin Normal University, Huaian, China; 2 College of Economics and Management & Research Centre for Soft Energy Science, Nanjing University of Aeronautics and Astronautics, Nanjing, China; Kwame Nkrumah University of Science and Technology, GHANA

## Abstract

Civil aviation carbon emission reduction is an inevitable requirement for achieving sustainable social development. Realizing the continuous expansion of air transportation scale while reducing the impact on the environment is particularly important. Therefore, it is necessary to accurately understand the relationship between civil aviation carbon emissions and the industry development. This study established a civil-aviation-pointed Tapio decoupling model to identify the decoupling state between transportation scale added and carbon dioxide emissions in China’s civil aviation sector. The index decomposition analysis method is further applied to decompose the factors influencing the changes in decoupling states. The empirical study generated three important findings. Firstly, the overall carbon emissions in the civil aviation sector are still growing, while the energy intensity has a tendency to fluctuate and decrease. Secondly, the relationship between carbon emissions and transport turnover is dominated by the expansive coupling, that is, the development of the civil aviation sector is still at the cost of the growth of energy consumption. Nevertheless, the overall decoupling stability is unstable, and the decoupling state is likely to be changed by many external factors. Thirdly, the energy intensity decoupling effect and industry structure decoupling effect are the main reasons for civil aviation carbon decoupling. Meanwhile, the improvement of national economic level during the research period is the dominant negative factor that restrains the carbon decoupling of the civil aviation sector.

## 1 Introduction

Global warming has become an important obstacle to the sustainable development of economy and society. How to effectively control and reduce the emission of greenhouse gases (GHGs), mainly carbon dioxide (CO_2_) emissions (accounting for approximately 77% of the GHGs), has gradually emerged as a major issue in the 21^st^ century [1]. As the basic carrier and strategic pilot industry of national economic and social development, transportation industry accounted for 24.34% of the total carbon emissions in 2021 [2]. Of which, civil aviation sector was the fastest growing mode of transportation in terms of carbon emissions, consuming more than 5 million barrels of oil per day [3]. From 2013 to 2019, the carbon emission of the global civil aviation transportation industry has exceeded 70% of the predicted value by the International Civil Aviation Organization (ICAO) [4, 5]. If unchecked, 25% of the world’s carbon emissions will come from the aviation industry by 2050 [6].

As for China, the civil aviation industry has played an effective role in stimulating China’s economic growth while developing rapidly. Its transportation revenue has increased from 0.37 billion yuan in 1980 to 1062.5 billion yuan in 2019 (occupied 1.08% of the Gross Domestic Product), yielding an average annual growth rate of 30.1% [7]. In the same period, the Revenue Ton Kilometers (RTK) has increased from 1.27 billion RTKs to 129.33 billion RTKs, with an average annual growth rate of 12.6%. This growth in civil aviation scale has, however, lead to increased CO_2_ emissions and significant environmental and climate concerns [8].

Considering the industry characteristic, the extremely high investment cost, long R&D and application cycle and ultra-high safety requirements in the civil aviation field make it the most difficult to achieve near zero emissions [9, 10]. Chinese government has introduced a series of emission reduction measures. For example, In January 2022, the Civil Aviation Administration of China (CAAC) issued the *14*^*th*^ *Five-year Special Plan for Green Development of Civil Aviation*, which defined the guiding ideology, basic principles, requirements and main tasks of civil aviation green development during the 14^th^ five-year plan period (from 2021 to 2025). It is clear that the development goal by 2025 is the carbon intensity will continue to decline, and the proportion of low-carbon energy consumption will continue to increase. In the long run, by 2035, China’s civil aviation should achieve carbon neutral growth [11].

In order to reach these ambitious targets, it is of great practical significance to analyze the impact of air transportation on the environment. However, focusing on revealing the relationship between the change of carbon emission and transportation development of civil aviation department has been barely discussed. Thus three questions are raised for China’s civil aviation, as follows: (1) Has China’s civil aviation decoupled from carbon emissions in the development process? From time series perspective, does this decoupling relationship have periodic characteristics? (2) What are the key factors influencing the change of the decoupling status between civil aviation carbon emissions and the industry development? (3) What are the differences of decoupling process and its driving factors among different time periods? And how to push the decoupling process in China’s civil aviation sector in the future? To reply the above three questions, this paper tries to establish a civil-aviation-pointed Tapio decoupling model to identify the decoupling state between transportation scale added and carbon dioxide emissions in China’s civil aviation sector from 1996 to 2015. And then index decomposition analysis (IDA) method is applied to decompose the factors influencing the changes in decoupling states.

Regarding remains of this paper, section 2 reviewed the relevant literatures. Section 3 introduces the decoupling model and decomposition method. Section 4 describes the data sources and processing. Section 5 discusses the results. Section 6 concludes the whole study and further provides the policy recommendations.

## 2 Literature review

According to the research by Liu et al. [10], China’s civil aviation carbon emission reduction pressure mainly comes from two aspects. On one hand, the ICAO adopted the international aviation carbon offset and emission reduction plan in 2016, requiring airlines to compensate for the carbon dioxide emissions of international flights that exceed the baseline. As such, the international routes of Chinese airlines will bear greater pressure on carbon emission reduction [11]. On the other hand, with the improvement of per capita income, the scale of civil aviation transportation in China will continue to expand, and the carbon emissions generated by aviation kerosene consumption will also continue to rise. Under the dual pressure of international and domestic emission reduction, policy makers hope that in the process of realizing the rapid development of civil aviation department, they can reduce the damage to the environment at the same time. Therefore, it is necessary to grasp the internal relationship between the carbon emissions of the civil aviation sector and the development of the industry from a macro perspective, so as to realize the green development of the civil aviation sector more effectively.

In the existing research, the mainstream methods to accurately describe the relationship between economic development and resource and environmental pressure are generally measured by EKC (environment Kuznet curve) and decoupling analysis [12]. EKC, coming from Kuznet curve, is a method that can usually be used for examining economic growth and energy/emission intensity with curve fitting. The representative study by Wang et al. [13] concluded that the existence of the EKC Hypothesis is established given an inverted U-shaped curve of CO_2_ emissions along with the increase of industrial output value. Meanwhile, the authenticity of the EKC hypothesis has also been questioned. Some scholars believe that environmental pollution caused by economic growth in different countries or regions has “heterogeneity” and “spatial correlation”, and different sample areas and econometric models will lead to different curve results. For example, Azlina et al. [14] examined the dynamic linkages among transport energy consumption, income and carbon dioxide emission in Malaysia during 1975 to 2011 using the EKC hypothesis. The results indicate that the inverted U-shape EKC hypothesis does not fully agree with the theory. The time duration and the annual data used for the present study do not seem to strongly validate the existence of EKC hypothesis in the case of Malaysia. In addition, EKC hypothesis is impossible to describe the correlation between economic growth and whether pollution emissions change synchronously.

Decoupling is another method, which calculates with the growth rate of economy and energy consumption or emission. In fact, the word “decoupling” first appeared in the field of physics, indicating the separation between two or more physical quantities with a response relationship [15]. The “decoupling” theory was first introduced into China’s environmental and economic research field by Zhang et al. [16]. By comparison with EKC, “decoupling”, with more specific conception and less calculation, can be understood and operated in an easy way. More and more studies have adopted this model to analyze the relationships among economy, energy use and emission [17–19]. Representative studies include Han et al. [20], which quantitatively analyzed the decoupling relationship between China’s economic growth and carbon emissions from 2000 to 2014. The results showed that China was in an expansionary negative decoupling state from 2002 to 2005, and was in a weak decoupling state from 2005 to 2014. Specifically, Liu et al. [8] pointed out that affected by phased policies, the economic development and carbon emissions have obviously phased characteristics. Therefore, it is of practical significance to study the stage changes of carbon emission decoupling state. Gao et al. [21] analyzed the decoupling relationship between economic growth and carbon emissions of 30 provinces in China from 1995 to 2017, the results showed that the decoupling status has experienced phased characteristics. And the decoupling states gradually moved from “expansive negative decoupling” during the “10^th^ five-year plan” period to “weak decoupling” or even “strong decoupling” during the “12^th^ five-year plan” period.

As described above, the decoupling analysis method can effectively describe the dynamic dependence between environmental pollution and economic growth. However, the reasons for the above dependence relationship cannot be identified. Existing studies pointed out that decomposition analysis methods can compensate for this defect and it can further analyze the effects of various drivers and provide reference for the formulation of energy conservation and emission reduction policies [22, 23]. Among which, structural decomposition analysis (SDA) and index decomposition analysis (IDA) are the two dominant methods applied in the environment field, and fruitful research results have been obtained. SDA is based on input-output table and has high requirements for the data size, which limits its application [24]. Compared with SDA method, due to the more flexible adoption and less data requirement, IDA method attained widespread use in the field of environment research [25]. Popular IDA methods include two indices: the Laspeyres index and the Divisia index. Ang [26] compared the existing various IDA methods, and proposed that Logarithmic Mean Divisia Index (LMDI) is better.

Therefore, the combination of decoupling analysis and decomposition analysis is not only helpful to identify the relationship between economic growth and carbon emissions, but also can explore the mechanism leading to the change in decoupling status. As such, many studies used the comprehensive method for empirical research. Representative examples include Dong et al. [27], whose study detected the decoupling relationship between carbon emission and economic growth through Tapio decoupling model, and further decomposed the influencing factors in decoupling relationship by IDA method. Raza and Lin [23] employed the decomposition and decoupling methods in driving factors of CO_2_ emissions from electricity generation for the years 1990–2019. Specifically, in the field of transportation, plenty of existing studies have been conducted to explore the driving factors in CO_2_ decoupling in transportation sector. The dominant factors influencing changes in transportation carbon decoupling can generally be grouped into two effects: the transportation activity effect and the energy use effect. For example, Kito et al. [28] combines the Tapio decoupling model and the LMDI method to analyze the relationship between transportation sector development and CO_2_ emissions. The result showed that transportation-intensity effect is the main factor that inhibits carbon decoupling in developed countries, while the energy-intensity effect is key to promote transportation carbon decoupling in developing countries.

However, few studies have focused on the driving factors of the evolution of civil aviation carbon decoupling. The civil aviation sector carbon emission mainly occurs in the stage of air transport. In order to grasp the relationship between carbon emission and industrial development of civil aviation sector and identify the contradiction between the development of civil aviation sector and the environment, it is necessary to analyze the decoupling status between air transport growth and carbon emission. Since 1996, China’s civil aviation sector has ushered in a stage of opening up competition and deepening reform, the transportation scale has expanded steadily and rapidly [11]. Furthermore, China’s economic development is generally planned every five-years, the development of civil aviation transport industry has obvious stage characteristics.

As such, this study established a civil-aviation-pointed Tapio decoupling model to identify the decoupling state between transportation scale added and carbon dioxide emissions in China’s civil aviation sector from 1996 to 2015. The index decomposition analysis (IDA) method is applied to decompose the factors influencing the changes in decoupling states. Compared with the existing studies, the complementally combination of IDA and decoupling methods in civil aviation sector can not only effectively identify the contradiction between air transportation development and environmental pollution, but also can explain the mechanism of dynamic evolution of decoupling state, which is conducive to targeted formulation of emission reduction policies.

## 3 Methodology

### 3.1 LMDI decomposition model

A large number of existing empirical studies on the application of IDA methods indicated that Logarithmic Mean Divisia Index (LMDI) method has better mathematical characteristics (such as zero value processing) and strong applicability [29, 30]. At the same time, the LMDI method has some other desirable properties in the context of decomposition analysis. Therefore, the LMDI technique is selected to construct the decomposition model in this paper.

The civil aviation carbon emissions in year t, *C*^*t*^, can be expressed as Eq (1):

$$\begin{array}{l} C^{t}=\sum_{i}C_{i}^{t}\\ \;=\sum_{i}\frac{C_{i}^{t}}{E_{i}^{t}}\cdot \frac{E_{i}^{t}}{E^{t}}\cdot \frac{E^{t}}{V^{t}}\cdot \frac{V^{t}}{Y^{t}}\cdot \frac{Y^{t}}{GDP^{t}}\cdot GDP^{t}\\ \;=\sum_{i}CI_{i}^{t}\cdot ES_{i}^{t}\cdot EI^{t}\cdot TI^{t}\cdot IS^{t}\cdot GDP^{t} \end{array}$$ (1)

On the left hand of Eq (1), *C* is the civil aviation carbon dioxide emissions; *t* is the study period. On the right hand of Eq (1), *i* represents the *i*th type of energy, *E* is the energy consumption volume of civil aviation sector. As such, *CI*_*i*_ = *C*_*i*_/*E*_*i*_ represents the carbon emission coefficient of the *i*th energy type; *ES*_*i*_ = *E*_*i*_/*E* denotes the proportion of the *i*th type energy consumption. Considering the factor that the carbon emission coefficient of a specific energy type does not change in a short term (invariant constant), *CI*_*i*_ and *ES*_*i*_ jointly represents the energy consumption structure of civil aviation sector [13]. *V* is the air transportation turnover, therefore *EI* = *E*/*V* denotes the energy consumption per unit transportation turnover, representing the energy intensity;*Y* is the transportation revenue of civil aviation sector, therefore $TI=\frac{V}{Y}$ denotes the transportation turnover per unit transportation revenue, reflecting the transportation intensity; *IS* =*Y*/*GDP* denotes the proportion of the civil aviation transportation revenue in the total gross domestic product, representing the industrial structure. According to the LMDI decomposition method, during the period of 0 to t, civil aviation carbon emission changed can be expressed as Eq (2):

$$\begin{array}{l} \Delta C=C^{t}-C^{0}\\ \;=\Delta C_{ci}+\Delta C_{es}+\Delta C_{ei}+\Delta C_{ti}+\Delta C_{is}+\Delta C_{gdp} \end{array}$$ (2)

The contribution of each influencing factors on the right side of Eq (2) can be calculated by Eqs (3A–3F).


$$\Delta C_{ci}=\{\begin{array}{l} 0,\;if\;C_{i}^{t}\times C_{i}^{0}=0;\\ \sum_{i}L(C_{i}^{t},C_{i}^{0})\ln(\frac{CI^{t}}{CI^{0}}),\;if\;C_{i}^{t}\times C_{i}^{0}\neq 0; \end{array}$$ (3A)

$$\Delta C_{es}=\{\begin{array}{l} 0,\;if\;C_{i}^{t}\times C_{i}^{0}=0;\\ \sum_{i}L(C_{i}^{t},C_{i}^{0})\ln(\frac{ES^{t}}{ES^{0}}),\;if\;C_{i}^{t}\times C_{i}^{0}\neq 0; \end{array}$$ (3B)

$$\Delta C_{ei}=\{\begin{array}{l} 0,\;if\;C_{i}^{t}\times C_{i}^{0}=0;\\ \sum_{i}L(C_{i}^{t},C_{i}^{0})\ln(\frac{EI^{t}}{EI^{0}}),\;if\;C_{i}^{t}\times C_{i}^{0}\neq 0; \end{array}$$ (3C)

$$\Delta C_{ti}=\{\begin{array}{l} 0,\;if\;C_{i}^{t}\times C_{i}^{0}=0;\\ \sum_{i}L(C_{i}^{t},C_{i}^{0})\ln(\frac{TI^{t}}{TI^{0}}),\;if\;C_{i}^{t}\times C_{i}^{0}\neq 0; \end{array}$$ (3D)

$$\Delta C_{is}=\{\begin{array}{l} 0,\;if\;C_{i}^{t}\times C_{i}^{0}=0;\\ \sum_{i}L(C_{i}^{t},C_{i}^{0})\ln(\frac{IS^{t}}{IS^{0}}),\;if\;C_{i}^{t}\times C_{i}^{0}\neq 0; \end{array}$$ (3E)

$$\Delta C_{gdp}=\{\begin{array}{l} 0,\;if\;C_{i}^{t}\times C_{i}^{0}=0;\\ \sum_{i}L(C_{i}^{t},C_{i}^{0})\ln(\frac{GDP^{t}}{GDP^{0}}),\;if\;C_{i}^{t}\times C_{i}^{0}\neq 0; \end{array}$$ (3F)

Of which, $C_{i}^{t}=CI_{i}^{t}\cdot ES_{i}^{t}\cdot EI^{t}\cdot TI^{t}\cdot IS^{t}\cdot GDP^{t}$, $L(C_{i}^{t},C_{i}^{0})=\left(C_{i}^{t}-C_{i}^{0}\right)/\left(\ln C_{i}^{t}-\ln C_{i}^{0}\right)$.

As such, the influencing factors of carbon emission changes during the period of 0 to *t* can be decomposed into 6 components, summarized in Table 1.

**Table 1:** Connotations of carbon emissions change influencing factors.

Factors	Connotation (during the period of 0 to *t*)
**Δ***C***_***ci***_**	The effect of carbon emission coefficient of different energy type on carbon emission change in civil aviation sector.
**Δ***C***_***es***_**	The effect of energy consumption structure change on carbon emission change in civil aviation sector.
**Δ***C***_***ei***_**	The effect of energy intensity on the civil aviation carbon emission changes.
**Δ***C***_***ti***_**	The effect of transportation intensity on the civil aviation carbon emission changes.
**Δ***C***_***is***_**	The effect of industrial structure on the civil aviation carbon emission changes.
**Δ***C***_***gdp***_**	The effect of social economic output on the civil aviation carbon emission changes.

### 3.2 Decoupling indicator

Based on the Tapio model [31], the measurements for identifying the relationship between carbon emissions and transportation turnover in China’s civil aviation sector can be expressed as Eq (4).

$$\varepsilon =\frac{\Delta C/C}{\Delta V/V}$$ (4)

where *ε* denotes the decoupling elasticity index between transport turnover of civil aviation sector and carbon emissions; Δ*C* is the change of the civil aviation carbon emissions during the studying period; Δ*V* is the change of the civil aviation transportation turnover during the studying period.

In order to further identify the drivers that leading to the change of the decoupling state, by combining Eq (2) and Eq (4) together, the decoupling state of civil aviation carbon emission from transportation turnover can be expressed as the sum of the following six components:

$$\begin{array}{l} \varepsilon =\frac{\Delta C/C}{\Delta V/V}\\ \;=\frac{\Delta C_{ci}/C}{\Delta V/V}+\frac{\Delta C_{es}/C}{\Delta V/V}+\frac{\Delta C_{ei}/C}{\Delta V/V}+\frac{\Delta C_{ti}/C}{\Delta V/V}+\frac{\Delta C_{s}/C}{\Delta V/V}+\frac{\Delta C_{gdp}/C}{\Delta V/V}\\ \;=\varepsilon_{ci}+\varepsilon_{es}+\varepsilon_{ei}+\varepsilon_{ti}+\varepsilon_{is}+\varepsilon_{gdp} \end{array}$$ (5)

Eq (5) shows that the decoupling state between carbon emissions of the Civil aviation sector and transportation turnover is decomposed into six effects, namely, carbon intensity decoupling effect (*ε*_*ci*_), the energy consumption structure decoupling effect (*ε*_*es*_), the energy intensity decoupling effect (*ε*_*ei*_), the transportation intensity decoupling effect (*ε*_*ti*_), the industrial structure decoupling effect (*ε*_*is*_), and the economic development decoupling effect (*ε*_*gdp*_).

Specifically, decoupling indexes (including *ε*, *ε*_*ci*_, *ε*_*es*_, *ε*_*ei*_, *ε*_*ti*_, *ε*_*is*_, and *ε*_*gdp*_) of China’s civil aviation can be divided into three types, i.e., the negative decoupling state, the decoupling state, and the connection state. In addition, these three states can be further divided into 8 categories, i.e., the expansive negative decoupling, the weak negative decoupling, the strong negative decoupling, the recessive decoupling, the weak decoupling, the strong decoupling, the expansive coupling, and the recessive coupling. The specific classification and basis are described in Table 2.

**Table 2:** Decoupling states and their classification.

Decoupling state		*ε*	Δ*C*	Δ*V*
**Negative decoupling**	Expansive negative decoupling	*ε*>1.2	Δ*C*>0	Δ*V*>0
	Weak negative decoupling	0<*ε*<0.8	Δ*C*<0	Δ*V*<0
	Strong negative decoupling	*ε*<0	Δ*C*>0	Δ*V*>0
**Decoupling**	Recessive decoupling	*ε*>1.2	Δ*C*<0	Δ*V*<0
	Weak decoupling	0<*ε*<0.8	Δ*C*>0	Δ*V*>0
	Strong decoupling	*ε*<0	Δ*C*<0	Δ*V*<0
**Connection**	Expansive coupling	0.8<*ε*<1.2	Δ*C*>0	Δ*V*>0
	Recessive coupling	0.8<*ε*<1.2	Δ*C*<0	Δ*V*<0

## 4 Data description

Since 1996, China’s civil aviation sector has ushered in a stage of opening up competition and deepening reform, the transportation scale has expanded steadily and rapidly [10]. However, affected by the COVID-19 epidemic, the carbon emissions and other relevant indicators of China’s civil aviation department are abnormal since the end of 2019. Considering the fact that China’s economic and civil aviation development policies and measures are generally planned every five years. The decoupling process of China’s civil aviation transport industry has obvious stage characteristics. Therefore, the research interval of this study focuses on the period from 1996 to 2015. And the research on carbon emissions in this period is divided into four stages (the 9^th^ five-year plan period, 1996-2000; the 10^th^ five-year plan 2001-2005; the 11^th^ five-year plan period, 2006-2010; the 12^th^ five-year plan period, 2011-2015).

The scope of this paper covers commercial civil aviation of China, including all the freight and passenger transport. Data on aviation transportation revenue and transportation turnover are mainly taken from the annual *Statistical Data on Civil Aviation of China* (NSBC, 1997-2016) [32]. The total air transportation turnover (RTK) is composed of passenger transportation turnover and cargo transportation turnover. The air transportation revenue also includes passenger and freight revenue (converted into 1985 price), excluding airport service and income.

As for the CO_2_ emissions, a “fuel-based” method is generally applied for the calculation. The data of CO_2_ emissions is estimated by multiplying the amount of aviation fuel and the emission coefficient. The CO_2_ emission coefficient is calculated using the combustion equation for fuels. The aviation kerosene accounts for the vast majority of aviation energy consumption (over 99%). Thus, this study only considers aviation kerosene, whose CO_2_ emission coefficient is 3.16 tons of CO_2_ emitted per ton of aviation fuel consumption [33]. Specifically, the scope of civil aviation CO_2_ emissions doesn’t include those related to airport operations. The descriptive statistics are summarized in Table 3.

**Table 3:** Statistical descriptions of the different civil aviation variables.

Indicator (Unit)		Mean	Std. D.	Max	Min
**Energy consumption**	million ton	7.48	7.05	25.04	0.66
**CO**_**2**_ **emission**	million ton	34.02	20.68	78.87	9.49
**Revenue ton kilometer**	10^4^ ton-kilometers	34.65	23.75	85.17	8.06
**Transportation revenue**	billion yuan	81.45	64.90	201.58	13.17

## 5 Results and discussions

### 5.1 Civil aviation carbon emission situation

Civil aviation carbon intensity is calculated by using the ratio of CO_2_ emissions to the corresponding transport turnover, generally reflecting the technical level of energy use. With the increasing demand for civil aviation transportation, the fleet size of airlines is also expanding, making China’s air transportation scale increase sharply. Meanwhile, with the continuous improvement of the operation level of airlines and the policy guidance of the government on carbon reduction, China’s civil aviation has achieved better performance in energy conservation and emission reduction. Thus leading to the continuous decline of the carbon emission intensity of the civil aviation sector.

**Fig 1:**
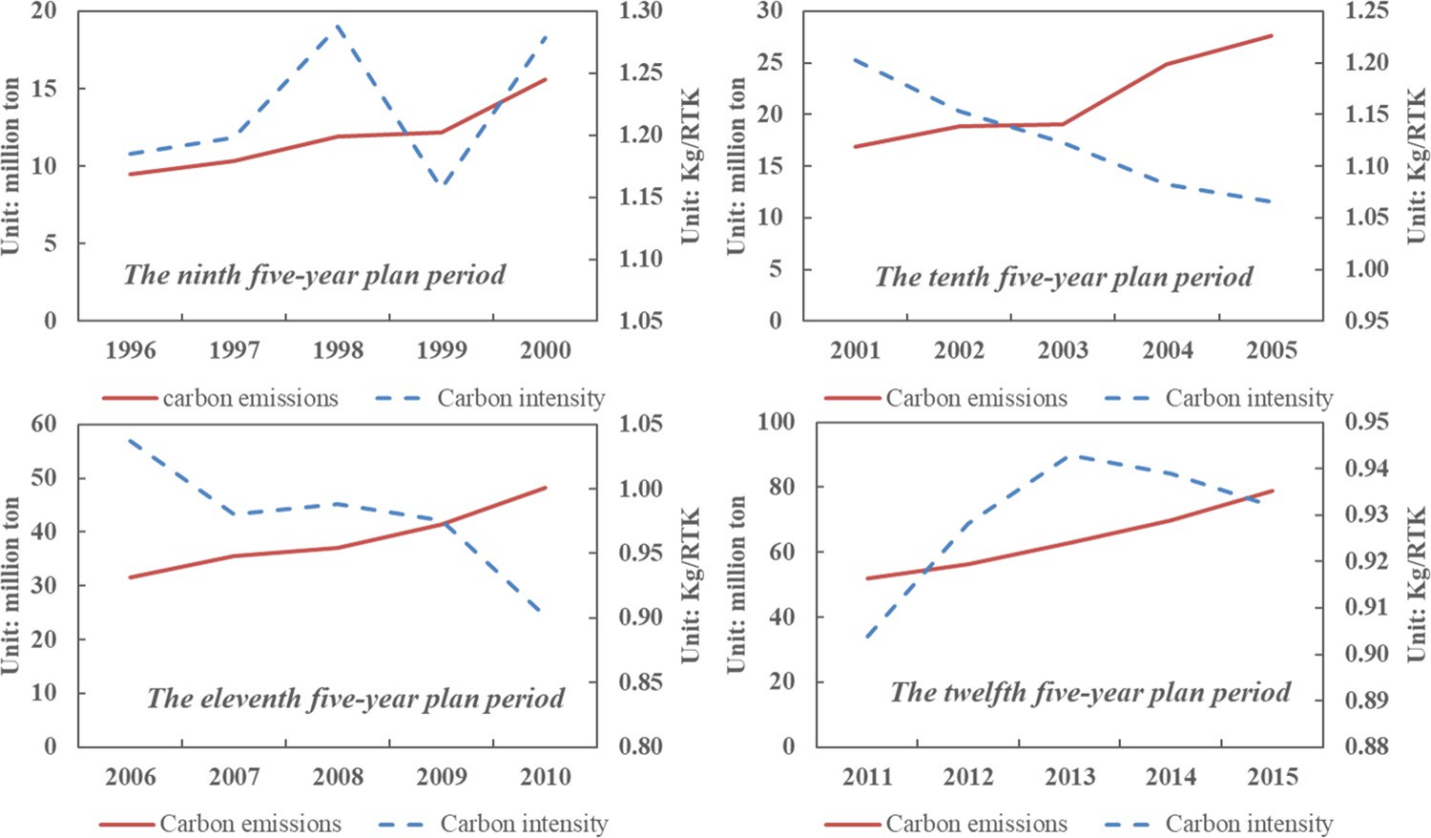
CO_2_ emissions and carbon emission intensity of China’s civil aviation, 1996-2015.

In the course of civil aviation transportation, carbon dioxide is produced due to consumption of jet fuel, which has an impact on the environment. As is shown in Fig 1, the carbon intensity of China’s civil aviation has experienced a fluctuated decreasing trend, decreasing from the highest 1.29 Kg/RTK in 1998 to 0.93 Kg/RTK in 2015. Except for the period of 1996-2000, influenced by the Asian financial crisis, the carbon intensity showed a slight upward trend. Due to the progress of energy use technology, during the 10^th^ and 11^th^ five-year periods, the carbon intensity decreased sharply. However, in the 12^th^ five-year plan period, the carbon intensity has a “up-down” trend, which indicates that the implementation of technology-based emission reduction has encountered a bottleneck. This is consistent with the research by Zhou et al. [33]. From the perspective of civil aviation carbon emissions, even though the decreasing trend of carbon intensity, the overall CO_2_ emissions are still on the rise, and the growth rate is getting bigger. The civil aviation sector should face up to the pressure of emission reduction and formulate targeted emission reduction countermeasures, so as to realize the continuous growth of transportation turnover and effectively control the increase of its carbon emissions.

In a certain period of time, air transportation cannot get rid of the dependence on jet fuel; jet fuel consumption will still increase with the increase of air traffic volume. The energy consumption and carbon emissions of the civil aviation industry are mainly concentrated in air transport and airport ground areas, of which air transport accounts for the vast majority. Therefore, it is of great practical significance to analyze the impact of air transport on the environment and reveal the relationship between the change of carbon emissions in the civil aviation sector and transport turnover.

### 5.2 Decoupling analysis

**Fig 2:**
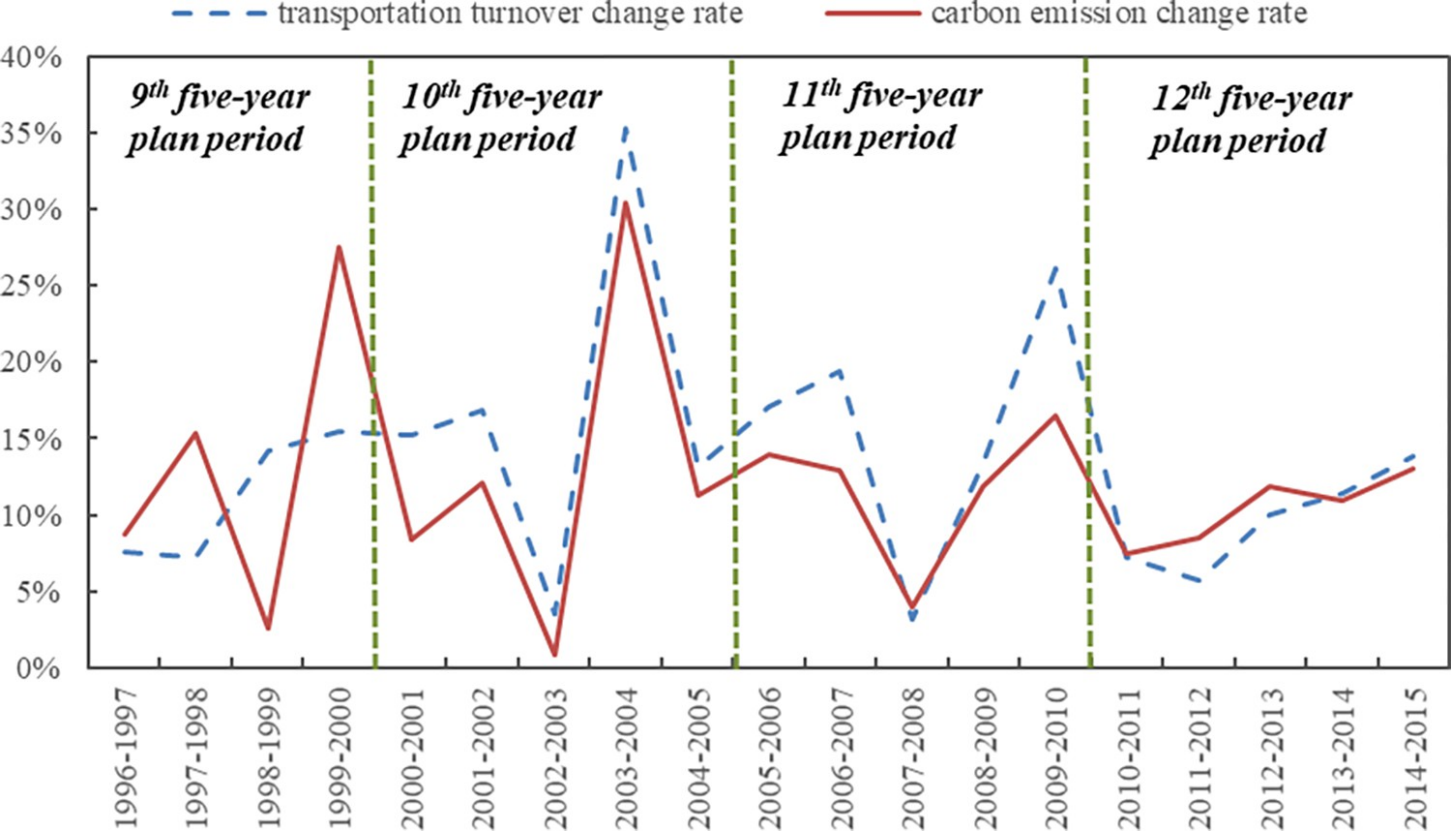
Change rates of civil aviation transportation scale and CO_2_ emissions, 1996-2015.

On the whole, it can be seen from Fig 2 that the change rate of transport turnover and the change rate of carbon emissions have basically the same trend, indicating the development of civil aviation sector cannot get rid of its impact on the environment.

Overall, the growth rate of air transportation turnover and carbon emissions has a downward trend. Specifically, during the four planning periods, the 9^th^ five-year plan period may be affected by the Asian financial crisis and the overall domestic economic level is not high. During the 10^th^ five-year plan period, the average annual growth rate of transportation turnover and carbon emissions of civil aviation is the highest, reaching 16.63% and 13.16% (see Fig 2), respectively. During the 12^th^ five-year plan period, the average annual growth rates of transportation turnover and carbon emissions were the lowest, at 10.2% and 11.05% (see Fig 2), respectively.

To further understand the internal relationship between the changes of carbon emission and transportation scale, based on Eq (4), the elasticity value of carbon emission decoupling of China’s civil aviation has been calculated and the decoupling status of each year was identified, as shown in Table 4.

**Table 4:** Decoupling status of carbon emissions in the civil aviation, 1996-2015.

Period		Δ*C*/*C*	Δ*V*/*V*	*ε*	Decoupling status
**The ninth five-year plan**	1996-1997	0.0868	0.0753	1.1527	Expansive coupling
	1997-1998	0.1529	0.0726	2.1061	Expansive negative decoupling
	1998-1999	0.0259	0.1413	0.1833	Weak decoupling
	1999-2000	0.2756	0.1544	1.7850	Expansive negative decoupling
	**1996-2000**	**0.6397**	**0.5197**	**1.2309**	**Expansive negative decoupling**
**The tenth five-year plan**	2000-2001	0.0839	0.1526	0.5498	Weak decoupling
	2001-2002	0.1204	0.1681	0.7162	Weak decoupling
	2002-2003	0.0080	0.0356	0.2247	Weak decoupling
	2003-2004	0.3040	0.3525	0.8624	Expansive coupling
	2004-2005	0.1132	0.1311	0.8635	Expansive coupling
	**2001-2005**	**0.6395**	**0.8505**	**0.7519**	**Weak decoupling**
**The eleventh five-year plan**	2005-2006	0.1395	0.1704	0.8187	Expansive coupling
	2006-2007	0.1293	0.1946	0.6644	Weak decoupling
	2007-2008	0.0395	0.0314	1.2580	Expansive negative decoupling
	2008-2009	0.1189	0.1335	0.8906	Expansive coupling
	2009-2010	0.1653	0.2608	0.6338	Weak decoupling
	**2006-2010**	**0.5306**	**0.7608**	**0.6974**	**Weak decoupling**
**The twelfth five-year plan**	2010-2011	0.0750	0.0724	1.0359	Expansive coupling
	2011-2012	0.0855	0.0569	1.5026	Expansive negative decoupling
	2012-2013	0.1181	0.1006	1.1740	Expansive coupling
	2013-2014	0.1090	0.1137	0.9587	Expansive coupling
	2014-2015	0.1299	0.1384	0.9386	Expansive coupling
	**2011-2015**	**0.5209**	**0.4749**	**1.0969**	**Expansive coupling**

From the calculation results, during the study period, the decoupling state of China’s civil aviation is dominated by expansive coupling. That is to say, the development of the civil aviation department is still at the cost of energy consumption growth, and coupling state maintains stable for a long time. During the four different five-year plan stages, the decoupling index values experienced an obvious downward trend, from 1.2309 in the 9^th^ five-year plan period down to 0.6974 in the 11^th^ five-year plan period. This means that the improvement of China’s civil aviation sector and emission reduction have greatly improved, thus alleviating the damage to the environment to a certain extent for the development of the industry. However, in the 12^th^ five-year plan period, the decoupling status presents the expansive coupling. In particular, from 2011 to 2012, the carbon emissions of civil aviation showed the expansive negative decoupling status, the growth of air transportation scale has greatly effect on the environment. During the 9^th^ five-year plan period, a similar situation occurred, and the overall decoupling status was also expansive negative decoupling. This can be generally attributed to the resisting of the Asian financial crisis in 1998 and the global financial crisis in 2008, when China’s civil aviation sector issued a series of stimulating development policy. These policies and measures effectively helped civil aviation rebound, meanwhile they also brought a series of negative effects. For example, the government tried to through high investment to boost domestic demand and airport or other infrastructure construction, however, some associated problems were ignored such as the route network planning and unreasonable capacity allocation and incomplete fleet structure and so on [34]. In fact, this is the continuation of the extensive development mode. During these time periods, according to the research by Liu et al. [8], the energy efficiency, carbon emission efficiency and operation efficiency of China’s civil aviation are generally very low. This should be the main reason for the long-term negative decoupling of the development of the civil aviation sector from the environmental impact.

China’s civil aviation carbon emissions and transportation turnover have been in a “weak decoupling” state for six years, i.e., 1998-1999, 2000-2003, 2006-2007 and 2009-2010. During these time periods, the growth rate of energy consumption was lower than that of air transportation, which is a relatively good state of decoupling air transport from carbon emissions. The main source of civil aviation carbon emissions is the consumption of aviation fuel. Considering the uniqueness of aviation fuel type, the progress of energy use technology and the improvement of operation efficiency are the main reasons for achieving weak decoupling of civil aviation carbon emissions. Specifically, during the 10^th^ five-year plan period, the average annual growth rate of investment in the civil aviation industry reached 18.7%. On the one hand, the energy use technology and energy efficiency were significantly improved by upgrading old fleets; on the other hand, the operational efficiency was effectively improved by strengthening exchanges and cooperation with other countries. This also explains the reason why civil aviation achieved weak decoupling state of carbon emissions during the 10^th^ five-year plan period.

The rest of the years were in a “expansive coupling” state, showing that China’s civil aviation energy consumption increased in parallel with air transport growth, and at roughly the same rate (see Fig 2). Judging from the development trend of air transport in recent years (during the “12^th^ five-year plan” period), there will be more growth connectivity in the coming period.

Considering that the development model of China’s civil aviation sector is changing from extensive to intensive, an effective measurement of the decoupling stability between carbon emissions of the civil aviation sector and transport turnover can provide a deeper understanding of the state characteristics of the energy dependence and environmental impact of the development of the civil aviation sector. Therefore, the decoupling stability coefficient is introduced in this paper, as shown in Eq (6).


$$S_{D}=\frac{1}{n}\sum_{t=1}^{n}\left|\frac{\varepsilon^{t+1}-\varepsilon^{t}}{\varepsilon^{t}}\right|$$ (6)

In Eq (6), *S*_*D*_ is the decoupling stability coefficient, *n* represents sample size, *ε*^*t*+1^ and *ε*^*t*^ represent the decoupling elastic values for *t*+1 period and *t* period, respectively. The larger the value of decoupling stability coefficient is, the easier the decoupling state is to change, and conversely, the more stable the decoupling state is. Bounded by 1, when *S*_*D*_>1, the decoupling state is likely to be repeated; When *S*_*D*_<1, the decoupling state is relatively stable.

**Fig 3:**
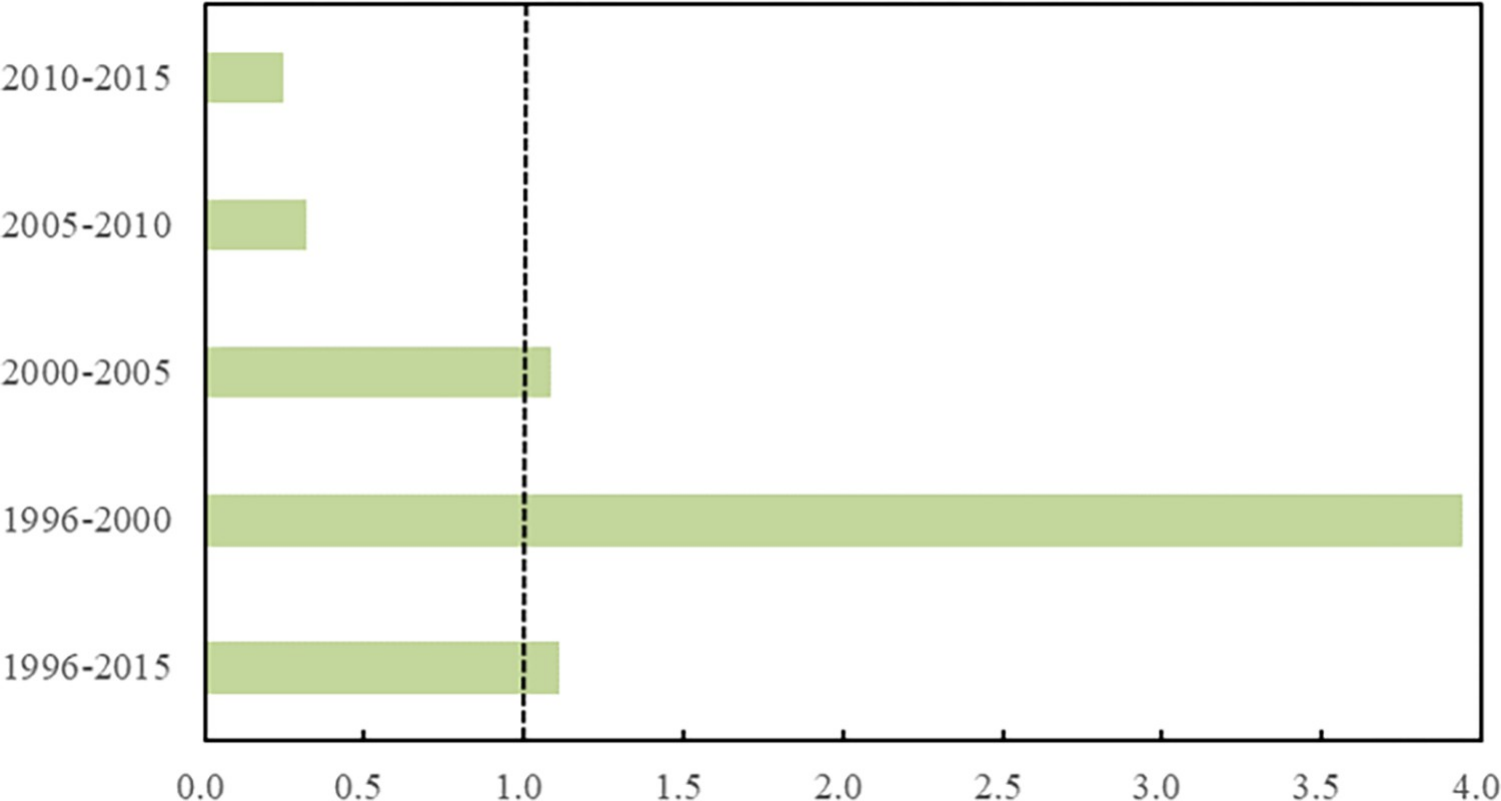
Decoupling stability index of carbon emission in civil aviation in different time periods.

Based on Eq (6), the decoupling stability coefficient of carbon emissions in the civil aviation sector at different stages can be obtained, as shown in Fig 3. Overall, the decoupling stability coefficient value during 1996 to 2015 is 1.11 (slightly greater than 1), indicating that the decoupling stability of the civil aviation sector is generally stable on the whole. During the “9^th^ five-year plan” period, the decoupling stability coefficient was 3.95, much larger than 1, and the stage stability was the worst. This may be due to the impact of the Asian financial crisis, the large fluctuation of energy prices, the instability of energy use behavior of civil aviation and the high empty load ratio of aircraft, making the decoupling state of carbon emissions of civil aviation sector unstable. During the “10^th^ five-year plan” period, the decoupling stability coefficient was 1.08, slightly higher than 1, indicating that the industrial development of China’s civil aviation sector was basically out of the impact of the financial crisis, and the transport turnover and carbon emissions basically increased simultaneously. During the “11^th^ five-year plan” and “12^th^ five-year plan” periods, the decoupling stability coefficient were only 0.32 and 0.24, indicating that the decoupling status of carbon emissions were relatively stable.

To sum up, there is a long away for China’s civil aviation achieving carbon emission decoupling. Many uncertainty factors may affect the progress in decoupling transportation growth from carbon emissions in China’s civil aviation, such as the macro environment, the contradiction between the civil aviation sector development and energy conservation and emissions reduction is still outstanding. As such, it is necessary to further explore the inner motivation of the civil aviation carbon emission decoupling.

### 5.3 Decomposition analysis

Based on the decomposition framework proposed above, the change of decoupling state is generally affected by energy structure, carbon emission coefficient, transportation intensity, energy intensity, industrial structure and the national economic development level. The decomposition results are shown in Table 5.

**Table 5:** Decoupling index and the influencing factors.

Period		*ε*_*ei*_	*ε*_*ti*_	*ε*_*is*_	*ε*_*gdp*_	*ε*_*tot*_
**The ninth five-year plan**	1996-1997	0.1477	0.1971	-0.4230	1.2312	1.1531
	1997-1998	1.0668	2.3581	-2.4365	1.1160	2.1044
	1998-1999	-0.7640	0.1411	0.2800	0.5263	0.1834
	1999-2000	0.7317	0.5107	-0.0513	0.5935	1.7844
**The tenth five-year plan**	2000-2001	-0.4196	0.6081	-0.1831	0.5442	0.5496
	2001-2002	-0.2624	-1.4214	1.8526	0.5477	0.7165
	2002-2003	-0.7616	2.4284	-4.1379	2.6960	0.2250
	2003-2004	-0.1186	0.0412	0.6277	0.3122	0.8625
	2004-2005	-0.1281	0.1972	-0.0684	0.8630	0.8639
**The eleventh five-year plan**	2005-2006	-0.1680	-0.0169	0.2551	0.7481	0.8184
	2006-2007	-0.3072	0.5400	-0.2922	0.7238	0.6645
	2007-2008	0.2553	2.0653	-4.0494	2.9880	1.2591
	2008-2009	-0.1031	0.7199	-0.4251	0.6986	0.8903
	2009-2010	-0.3263	-0.1100	0.6585	0.4117	0.6338
**The twelfth five-year plan**	2010-2011	0.0347	-0.2777	0.0056	1.2734	1.0359
	2011-2012	0.4879	0.2500	-0.5859	1.3498	1.5015
	2012-2013	0.1661	1.1708	-0.9429	0.7800	1.1741
	2013-2014	-0.0392	0.3514	-0.0062	0.6527	0.9587
	2014-2015	-0.0575	0.3338	0.1929	0.4696	0.9387
	**Mean**	**-0.0298**	**0.5309**	**-0.5121**	**0.9751**	**0.9641**

**Fig 4:**
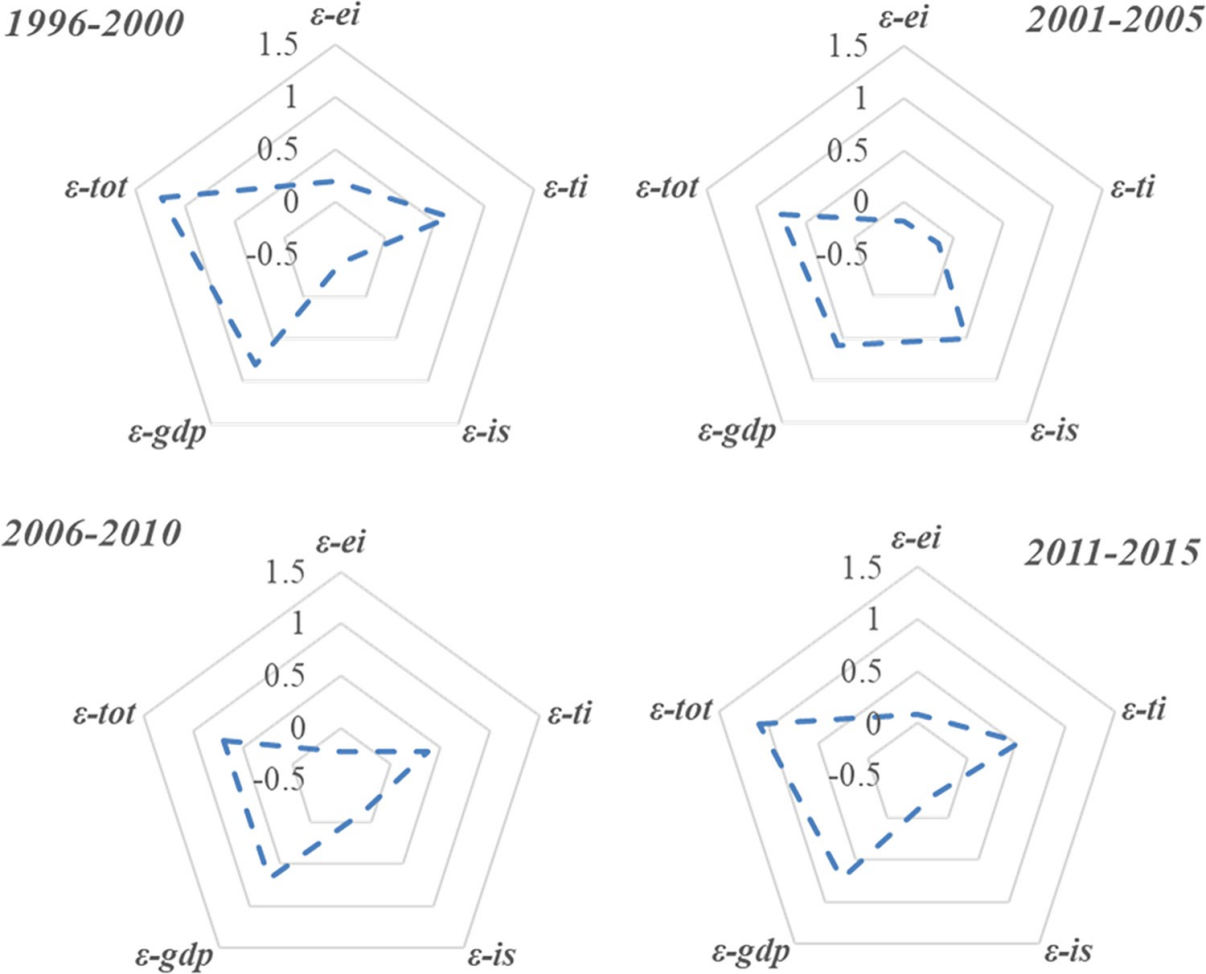
Influencing factors of carbon emission decoupling in civil aviation, 1996-2015.

Specifically, when the energy consumption structure of civil aviation sector changes, both *CI*_*i*_ and *ES*_*i*_ will change because of the different carbon emission coefficients of different energy types. However, due to the particularity of energy consumption in the civil aviation sector (the type of energy consumption is single and remains unchanged for a long time), both Δ*C*_*ci*_ and Δ*C*_*es*_ should be zero, leading to no effect on the change of carbon emission. In the future, development of alternative fuel is an important trend for the civil aviation sector. Considering the phased characteristics of civil aviation development, this study further analyzed the influence of different influencing factors in different stages (shown in Fig 4).

**Fig 5:**
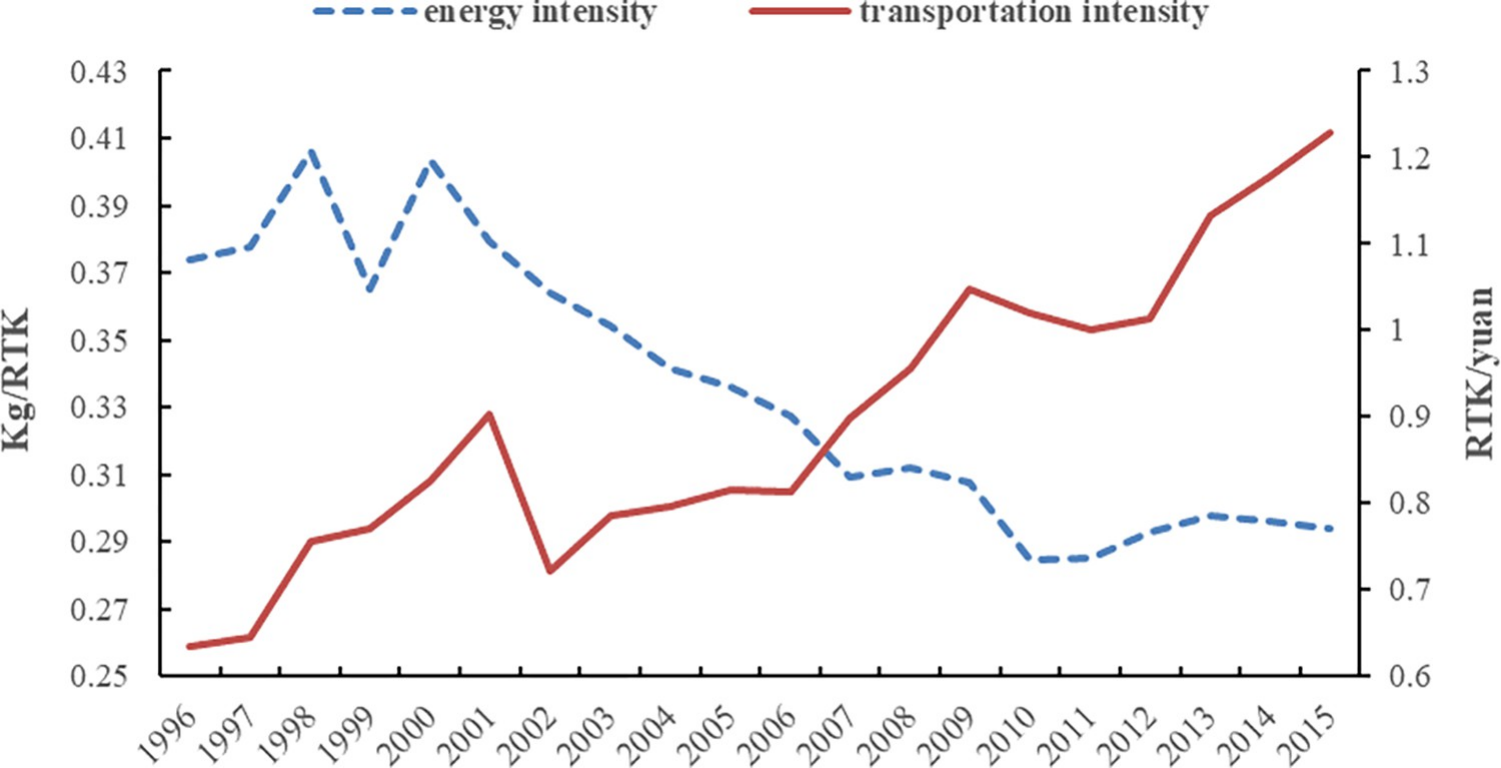
Changes in energy intensity and transportation intensity, 1996-2015.

It can be seen from Table 5 that the overall average impact value of energy intensity decoupling effect (*ε*_*ei*_) is -0.0298. This indicates that energy intensity help promote the trend of decoupling of carbon emission in the civil aviation sector. The energy intensity decoupling effect refers to the impact of the change of energy consumption per unit transportation turnover on the decoupling state of the civil aviation sector. If other factors remain unchanged, the decrease of energy intensity reflects that energy efficiency and the corresponding carbon emission output benefit are improving, which effectively promotes carbon emission decoupling. As shown in Fig 5, the energy intensity of the civil aviation sector has a downward. Specifically, *ε*_*ei*_ played the dominant role in contributing to carbon emission decoupling in the “10^th^ five-year plan” and “11^th^ five-year plan” periods, and the influencing values are -0.1841 and -0.2296, respectively. However, *ε*_*ei*_ did not promote carbon emissions decoupling during the “9^th^ five-year plan” and “12^th^ five-year plan” period, with contribution values of 0.1892 and 0.0804, respectively. It can be verified from Fig 5 that the energy intensity of the civil aviation sector fluctuates and rises in these two periods, especially from 1996 to 2000. This may be due to the Asian financial crisis (1996-1998), the civil aviation passenger and cargo transport demand cuts, resulting in a decline in load factor. Thus, leading to the increase of energy intensity. It should also be noted that since 2011, the energy intensity has experienced a “up-down” trend. This signifies that the advanced technologies that promote the decline of energy intensity has met bottlenecks, and its corresponding carbon decoupling index shows an upward trend. For the civil aviation department, the space to improve energy efficiency through technological progress is becoming gradually limited [7]. That is to say it has become increasingly difficult to realize the carbon emission decoupling through technological progress.

Transportation intensity decoupling effect (*ε*_*ti*_) refers to the influence of the change of air transportation activity on the decoupling state of carbon emissions. It can be seen from Table 5 that the overall average impact value of *ε*_*ti*_ is 0.5309. This indicates that transportation intensity inhibited the trend of decoupling of carbon emission in the civil aviation sector. From different time periods, *ε*_*ti*_ played the significant role in contributing to carbon emission decoupling in the “10^th^ five-year plan” (see Fig 4). However, *ε*_*ti*_ did not promote carbon emissions decoupling in other years. As shown in Fig 5, the overall transportation intensity has an upward trend, and the values of transportation intensity effect are greater than 0 in most of the years. On the premise of keeping other influencing factors unchanged, the improvement of transport intensity means that the transportation activity of civil aviation sector is strengthened, thus inhibiting the decoupling process of carbon emissions. In fact, there is a strict positive correlation between civil aviation carbon emissions and air transport scale. Transportation revenue is an accessory of the transportation process. Compared with the rapid growth of transportation turnover, the change rate of transportation revenue is relatively small. Therefore, the impact of transportation intensity is more affected by the change of transportation turnover, which also explains why the transportation intensity effect can inhibit the decoupling of carbon emissions.

Industrial structure decoupling effect (Δ*C*_*is*_) is another important fact that promotes the civil aviation carbon emission decoupling, which average impact value is -0.5121. The Δ*C*_*is*_ refers to the impact of the civil aviation transportation revenue proportion in GDP on the decoupling progress of carbon emissions. The transportation revenue of the civil aviation sector accounts for about 0.65% of GDP and fluctuates. There was an obvious downward trend in 1998-2001 and 2007-2009, indicating that the development of the civil aviation sector is very sensitive to the international economic environment. As shown in Table 5, the role of the civil aviation in the whole national economy is to promote the carbon decoupling of the civil aviation department. Therefore, we are reminded to dynamically grasp the development trend of the international economy, adjust the development plan of the civil aviation department in time, and further highlight the role of Δ*C*_*is*_ in promoting the carbon emission decoupling of civil aviation department.

Economic development decoupling effect (*ε*_*gdp*_) refers to the impact of the national economic level changes on the decoupling relationship between transport turnover and carbon emissions in the civil aviation sector. As shown in Table 5, the development of GDP during the research period is the dominant factor that restrains the carbon emission decoupling of the civil aviation sector. Benefits from the increasingly prosperous economy, people are willing and able to choose more comfortable, safe and convenient modes of air transportation, whereas the perfection of the transportation capacity of airlines has a far-reaching impact on the transportation scale and carbon emission. The increasing national income level continuously stimulated the demand for civil aviation transportation services. This may explain the negative role that *ε*_*gdp*_ played in the promoting of civil aviation carbon emission decoupling. Nevertheless, the impact degree of the economic development on the decoupling contribution rate is gradually reduced from 96.8% in the “11^th^ five-year plan” period to 66.7% in the “12^th^ five-year plan” period. This situation sends a positive signal that good economic environment and civil aviation carbon emission reduction can be achieved simultaneously in the future.

## 6 Conclusion

With the dramatically expanding of air transportation demand, the civil aviation sector will become a major source of CO_2_ emissions. In response to this global challenge, China’s civil aviation industry has embarked on a series of low-carbon development initiatives. In order to grasp the relationship between the carbon emissions of the civil aviation sector and the development of the industry, this study analyzes the decoupling status and further identifies the factors influencing the progress in decoupling transportation growth from carbon emissions during the period of 1996-2015. The empirical study generated three important findings.

Firstly, the overall CO_2_ emissions in the civil aviation sector are still growing, while the energy intensity has a tendency to fluctuate and decrease. With the increasing demand for civil aviation transportation, the fleet size of airlines is also expanding, making China’s air transportation scale increase sharply. Specifically, due to the progress of energy use technology, during the 10^th^ and 11^th^ five-year plan periods, the carbon intensity decreased sharply. However, in the 12^th^ five-year plan period, the carbon intensity has a “up-down” trend, which indicates that the implementation of technology-based emission reduction has encountered a bottleneck. China’s civil aviation should strengthen the policy guidance on carbon reduction to achieve better performance in energy conservation and emission reduction. For example, airlines can reduce the demand for air transport by levying carbon taxes, thus decreasing the carbon emissions.

Secondly, in order to explore the relationship between carbon emissions and the industry development, this paper conducts decoupling analysis on transport turnover and carbon emissions in the civil aviation sector. The relationship between carbon emissions of China’s civil aviation sector and transport turnover is dominated by expansive coupling status, that is, the development of the civil aviation sector is still at the cost of the growth of energy consumption. During the four different five-year plan stages, the decoupling index values experienced an obvious downward trend. This means that the improvement of China’s civil aviation sector and emission reduction have greatly improved, thus alleviating the damage to the environment to a certain extent for the development of the industry. However, the overall decoupling stability of the civil aviation sector’s carbon emissions is unstable, and the decoupling state is likely to be changed by many external factors. As such, it is necessary to further explore the motivation of the civil aviation carbon emission decoupling.

Thirdly, decomposition analysis discovered that the change of decoupling state is generally affected by energy structure, carbon emission coefficient, transportation intensity, energy intensity, industrial structure and the national economic development level. Of which, the energy intensity decoupling effect and industry structure decoupling effect are the main reasons for carbon decoupling. Specifically, energy intensity decoupling effect has only played the dominant role in contributing to carbon emission decoupling in the “10^th^ five-year plan” and “11^th^ five-year plan” periods. It has become increasingly difficult to realize the carbon emission decoupling through technological progress. Airlines need to timely adjust the fleet structure including retiring old aircrafts and upgrading new aircrafts, and match proper aircraft types with the flight ranges to decrease energy intensity. Meanwhile, the development of national economic during the research period is the dominant negative factor that restrains the carbon emission decoupling of the civil aviation sector. The increasing national income level continuously stimulated the demand for civil aviation transportation services. Nevertheless, the impact degree of the economic development on the decoupling contribution rate experienced a decreasing trend. This situation sends a positive signal that good economic environment and civil aviation carbon emission reduction can be achieved simultaneously in the future.

## Supporting information

S1 File (DOCX)
