# Supplementary material for: What drives the decoupling progress of China’s civil aviation transportation growth from carbon emissions? A new decomposition analysis
Source: PLoS One. 2023 Mar 6;18(3):e0282025. doi: 10.1371/journal.pone.0282025 (PMC9987793; doi:10.1371/journal.pone.0282025)
Supplement: S1 File — (DOCX) [file pone.0282025.s001.docx]

**What drives the decoupling progress of China’s civil aviation transportation growth from carbon emissions? A new decomposition analysis**

**Supporting Information**

**Introduction**

This supporting information file contains the compilation of **data processing** (Text S1), and **data analysis** (Text S2). Data to generate the figures in the main paper and Supporting Information are also included.

**Text S1. Data processing.**

The total air transportation turnover (*V*) is composed of passenger transportation turnover and cargo transportation turnover. The data of CO_2_ emissions (*C*) is estimated by multiplying the amount of aviation fuel (*E*) and the emission coefficient. This study only considers aviation kerosene; whose CO_2_ emission coefficient is 3.16. The scope of civil aviation CO_2_ emissions doesn’t include those related to airport operations. The air transportation revenue (*Y*) includes passenger and freight revenue, excluding airport service and income. The real GDP and transportation revenue were deflated by the consumer price index in 1985 to eliminate the price effect. Taking air transportation revenue (*Y*) as an example, the deflation is shown as follows:

$Y^{b}=dY^{n}$ (S1)

$d=\frac{1}{\prod_{m=b+1}^{n} \left(p^{m}/100\right)}$ (S2)

where $Y$ represents the revenue added. The superscript $b$ represents the value added in the base year price, and the superscript $n$ refers to the value added at current prices. $d$ represents the deflator derived from consumer price index $p^{m}$. $p^{m}$ refers to the chained price index (previous year=100) of the $m$th year, ranging from the base year to the $n$th year.

All the raw data were presented in Table S1. Table 3 in the manuscript contains the descriptive statistics of the raw data.

**Table S1:** The raw data related to calculation and results analysis in this paper.

|  | **Carbon emission （C，million ton）** | **Energy consumption （E，million ton）** | **Air transportation turnover （V，10^4^ ton-kilometers）** | **Air transportation revenue （Y，billion yuan）** | **Gross domestic product （GDP，billion yuan）** |
| --- | --- | --- | --- | --- | --- |
| **1996** | 9.4926 | 3.0135 | 8.0608 | 12.6924 | 20141.7596 |
| **1997** | 10.3169 | 3.2752 | 8.6677 | 13.4552 | 22014.3458 |
| **1998** | 11.8938 | 3.7758 | 9.2974 | 12.3061 | 23738.8058 |
| **1999** | 12.2022 | 3.8737 | 10.6113 | 13.7713 | 25547.6638 |
| **2000** | 15.5652 | 4.9413 | 12.2501 | 14.8281 | 27701.6587 |
| **2001** | 16.8704 | 5.3557 | 14.1192 | 15.6338 | 30000.9844 |
| **2002** | 18.9023 | 6.0007 | 16.4927 | 22.8837 | 32725.6942 |
| **2003** | 19.0537 | 6.0488 | 17.0795 | 21.7437 | 36006.5690 |
| **2004** | 24.8463 | 7.8877 | 23.0999 | 29.0375 | 39637.8458 |
| **2005** | 27.6594 | 8.7808 | 26.1272 | 32.0485 | 44120.9002 |
| **2006** | 31.5169 | 10.0054 | 30.5798 | 37.6112 | 49713.9012 |
| **2007** | 35.5916 | 11.2989 | 36.5299 | 40.7023 | 56754.5802 |
| **2008** | 36.9982 | 11.7455 | 37.6765 | 39.3939 | 62222.6957 |
| **2009** | 41.3964 | 13.1417 | 42.7073 | 40.7772 | 67956.0187 |
| **2010** | 48.2391 | 15.3140 | 53.8449 | 52.7950 | 75055.3766 |
| **2011** | 51.8580 | 16.4629 | 57.7443 | 57.7273 | 82035.4405 |
| **2012** | 56.2914 | 17.8703 | 61.0322 | 60.1867 | 88313.1486 |
| **2013** | 62.9409 | 19.9812 | 67.1723 | 59.2619 | 95113.2610 |
| **2014** | 69.8034 | 22.1598 | 74.8116 | 63.5449 | 102056.5291 |
| **2015** | 78.8713 | 25.0385 | 85.1650 | 69.2651 | 108486.0904 |

**Text S2. Data analysis.**

Fig.1 in the manuscript is the descriptive CO_2_ emissions and carbon emission intensity of China’s civil aviation during the period of 1996-2015. The data of CO_2_ emissions can be collected from Table S1; the data of carbon emission intensity can be obtained by dividing carbon emissions (*C*) by transportation revenues (*Y*).

Fig.2 in the manuscript is the descriptive change rates of civil aviation transportation scale and CO_2_ emissions during the period of 1996-2015. The change rates of CO_2_ emissions and transportation scale can be calculated using the data of Carbon emission (*C*) and Air transportation turnover (*V*) in Table S1.

The results in Table 4 have been clearly introduced in the manuscript.

Fig.3 in the manuscript is the descriptive decoupling stability index of carbon emission in civil aviation in different time periods. The decoupling stability coefficient can be calculated by using the equation of $S_{D}=\frac{1}{n}\text{ }\sum_{t=1}^{n} \left|\frac{\varepsilon^{t+1}-\varepsilon^{t}}{\varepsilon^{t}}\right|$.

Table 5 in the manuscript presents the decoupling index and the influencing factors. And the calculation process has been clearly introduced in the Methodology part.

Fig.4 in the manuscript is the descriptive influencing factors of carbon emission decoupling in civil aviation in different stages. Using the calculation steps introduced in the Methodology part, the effects of each influencing factor were calculated in four different stages, respectively.

Fig.5 in the manuscript is the descriptive changes in energy intensity and transportation intensity during the period of 1996-2015. The energy intensity can be obtained by dividing aviation fuel (*E*) by air transportation turnover (*V*); the transportation intensity can be obtained by transportation turnover (*V*) by transportation revenues (*Y*).
